# Electrophysical Properties and Heat Capacity of Activated Carbon Obtained from Coke Fines

**DOI:** 10.3390/molecules28186545

**Published:** 2023-09-09

**Authors:** Aigul T. Ordabaeva, Zainulla M. Muldakhmetov, Sergey V. Kim, Shuga B. Kasenova, Zhenisgul I. Sagintaeva, Arstan M. Gazaliev

**Affiliations:** 1 Institute of Organic Synthesis and Chemistry of Coal of Kazakhstan Republic, Alikhanov Str., 1, Karaganda 100000, Kazakhstan; iosu.rk@mail.ru (Z.M.M.); vanquishv8@mail.ru (S.V.K.); gazaliyev51@mail.ru (A.M.G.); 2 Laboratory of Thermochemical Processes, Zh. Abishev Chemical-Metallurgical Institute, Karaganda 100009, Kazakhstan; kasenovashuga@mail.ru (S.B.K.); kai_sagintaeva@mail.ru (Z.I.S.)

**Keywords:** activated carbon, heat capacity, electrical resistance, the relative permittivity

## Abstract

This paper studies the dependence of the specific heat capacity (C_p_) of activated carbon obtained by the activation of coke fines on temperature (T, K) and the dependence of electrical resistance (*R*, Om) on temperature (T, K). In the course of the work, it was found that in the temperature range of 298.15–448 K on the curve of dependence C_p_ − *f*(T) at 323 K there is a jump in heat capacity, associated with a phase transition of the second kind. Measurements of the temperature dependence of electrical resistance on temperature were also carried out, which showed that activated carbon in the temperature range of 293–343 K exhibits metallic conductivity, turning into a semiconductor in the temperature range of 343–463 K. The calculation of the band gap showed that the resulting activated carbon is a semiconductor with a moderately narrow band gap. The satisfactory agreement of the phase transition temperatures on the curves of the temperature dependences of the heat capacity on temperature (323 K) and on the curves of the dependences of electrical resistance and the relative permittivity on temperature (343 K) indicates the nature of this phase transition, i.e., at a temperature of 323 K, the change in heat capacity is associated with the transition from semiconductor conductivity to metallic.

## 1. Introduction

Activated carbon has a number of physicochemical properties and is therefore one of the most common materials for the production of capacitors, including supercapacitors [1,2]. As an electrode material, activated carbon has a number of advantages that make it attractive for use in electronic devices. Activated carbon has a very high surface area due to its microporous structure and numerous pores. This provides it with a large area for the adsorption of electrolyte ions, which leads to an increase in the capacitance of the capacitor. The high electrical conductivity of activated carbon contributes to the efficient transport of electrons in the charging and discharging processes. In addition, activated carbon has chemical stability, which ensures a long service life of the electrode and reliable operation of the capacitor. Activated carbon is a relatively inexpensive material, which makes it economically attractive.

However, it is important to note that activated carbon is not the only possible material for capacitor electrodes, and there are other materials that are also used and investigated in this field. The choice of material depends on the specific requirements and characteristics of the capacitor for a particular application. The variety of starting materials and the conditions of production make it possible to obtain electrode materials for energy storage devices with certain characteristics. Plant-based materials are an excellent material for producing electrode materials with high electrical characteristics. For example, in Lobato-Peralta et al. [3] it has been shown that the impregnation of corn husks in K_2_CO_3_ solution and activation in the temperature range of 500–800 °C leads to the formation of activated carbon with nanoscale pores, a specific surface area exceeding 1000 m^2^/g, a specific capacity of 269 F/g and an energy density of approximately 10 W·h/kg. The authors note that the assembled supercapacitor using the resulting activated carbon showed stable operation during 20,000 charge/discharge cycles with minimal loss of the initial charge. Activated carbon with a similar specific capacity of 268 F/g was obtained by Tu et al. [4] by carbonizing American ginseng waste (AGWR) in a tubular furnace at 350 °C for 2 h, followed by two-stage activation of the resulting material in a mixture with KOH, first at 350 for 1 h, and then at 800 for 2 h. However, in the latter case, the specific surface area of the activated carbon obtained was 2187 m^2^/g and the energy density was 18.6 W·h/kg with minimal losses of the initial energy during 10,000 charging–discharging cycles.

Carbonation and the subsequent activation of various types of plant raw materials provide the possibility of obtaining carbon materials with a high value of specific surface area [5].

Hu et al. [6] studied the possibility of obtaining varnished wood activated by activation in a pyrolysis autoclave in a nitrogen atmosphere and found that the sample obtained at 600 °C has a specific surface area of 1419.46 m^2^/g, a specific capacity of 354 F/g and exhibits excellent preservation of the initial energy over 10,000 charging–discharging cycles.

Nazhipkyzy et al. [7] showed that an autoclave treatment of mixtures of pine and elm sawdust with KOH solution at 120 °C for 2 h and subsequent carbonation of the resulting material in the temperature range 700–900 °C for 1 h in an argon atmosphere allows activated carbons to be obtained with specific capacity values of 147 F/g and 114 F/g with energy densities of 26.0 and 22.1 W·h/kg.

In addition to raw materials of plant origin, electrode materials for energy storage devices can be obtained from anthracite. At the same time, the activated carbons obtained, as well as in the case of activated carbons obtained from vegetable raw materials, are not inferior in some characteristics and even surpass commercial activated carbons. For example, activated carbons obtained by Piñeiro-Prado et al. [8] impregnated with Spanish anthracite with a KOH solution, when heated to 750 °C for 2 h, have a specific surface area of 3310 m^2^/g, a specific capacity of 40 F/g and an energy density of 10.9 W·h/kg.

The activation of a fine fraction of natural Morocco anthracite mixed with KOH at 850 °C in a nitrogen stream for 1 h allowed Boujibar et al. [9] to obtain activated carbon with a specific surface area of 2934 m^2^/g, a specific capacity of 42 F/g and a specific energy of 72.51 W·h/kg.

Ding et al. [10] used carbonation of anthracite (extracted in Inner Mongolia) at 800 °C in a nitrogen atmosphere for 3 h and the subsequent activation of the resulting material with a KOH solution, also at 800 °C in a nitrogen atmosphere, which obtained activated carbon with a specific surface area of 1695.1 m^2^/g and a specific capacity 85.2 F/g.

Shale pyrolysis products can also be a promising raw material for the production of materials for supercapacitors. Thus, Xiong et al. [11], using microwave separation and chemical activation, obtained activated carbon with a specific surface area of 1478 m^2^/g and a specific capacity of 185 F/g, preserving capacitance characteristics for 5000 cycles.

Scientific research in the field of the development of efficient energy storage is constantly evolving, and there are other materials that are also being investigated and can be applied in supercapacitors [12]. Among carbon materials, graphene should also be noted, which, due to a number of unique physicochemical properties, has a wide range of applications in various electronic devices [13,14,15].

Electropolymerization of polyaniline film on graphene paper, performed by Wang et al. [16], led to the formation of an electrode material with a specific capacity of 233 F/g.

Carbon nanotubes have a structure similar to graphene, but form tubular structures. They have high mechanical strength and electrical conductivity, which makes them attractive electrode materials for supercapacitors [17,18,19,20]. The electrical characteristics of electrodes using carbon nanotubes are impressive, so Bravio et al. [21] using chemical synthesis obtained a nanocomposite material consisting of polyaniline and carbon nanotubes, which demonstrated a specific capacity of 1744 F/g and a specific energy of 485 W·h/kg.

Metal oxides can also be used as electrodes for capacitors due to good chemical stability and high capacitance characteristics. Depending on the production conditions, it is possible to obtain electrodes with different electrical parameters [22,23].

The synthesis of compounds of metallic oxides with various polymers and carbon nanomaterials is another interesting area of research. Such hybrid electrodes can combine the advantages of both types of materials and improve the characteristics of the capacitor [24,25]. Metallic oxides such as rutile (TiO_2_), manganese dioxide (MnO_2_), nickel oxide (NiO) and others have a good ability to intercalate and deintercalate ions, which provides a higher capacity compared to pure carbon materials [26].

The combination of various types of physico-chemical processes also makes it possible to obtain nanoscale materials for supercapacitors. So, by a combination of sol–gel synthesis, hydrothermal treatment and annealing, Bazaluk et al. [27] obtained an ultrafine superparamagnetic ‘Core/Shell’ γ-Fe_2_O_3_/Defective α-Fe_2_O_3_ composites with a specific capacity of 124 F/g.

Of course, each combination of polymers and carbon nanomaterials with metal oxides can give different results based on the properties of the materials used and methods of their synthesis. Research in this area is actively conducted, and new hybrid materials continue to be developed to improve the characteristics of supercapacitors.

In this paper, studies have been conducted to study the electrophysical characteristics and heat capacity of activated carbon (AC) obtained from coke fines (CF) by the method presented in [28]. The possibility of using activated carbon obtained in previous studies as a material for capacitors has been evaluated. 

Determination of the heat capacity of activated carbon allows an assessment of the nature of the influence of temperature on its adsorption properties [29]. Modeling of processes based on the regularities of the influence of heat capacity on kinetics, thermodynamics and the stability of adsorption and desorption processes gives an idea of the possibility of developing technologies for adsorbed natural gas [30].

## 2. Results and Discussion

### 2.1. Heat Capacity Measurement

Table 1 presents the data on measuring the heat capacity C_p_ [J/(g · K)] of a sample of the starting material coke fines (CF) and the obtained activated carbon (AC) in the range of 298.15–448 K.

It can be seen from the experimental data in Table 1 that a monotonous increase in heat capacity is observed for CF. Based on the data obtained, the equation of the temperature dependence of the heat capacity C_p_ [J/(g · K)] for CF will have the form:

C_p_ = (0.196 ± 0.011) + (1.98 ± 0.11) × 10^−3^T + (0.021 ± 0.001) × 10^5^T^−2^ (1)

For the temperature intervals under consideration, the value of the average random error was used to determine the error of the coefficients in the dependence equations C_p_~f(T).

The graphical dependencies C_p_~f(T) for coke fines (CF) and activated carbon (AC)

**Figure 1:**
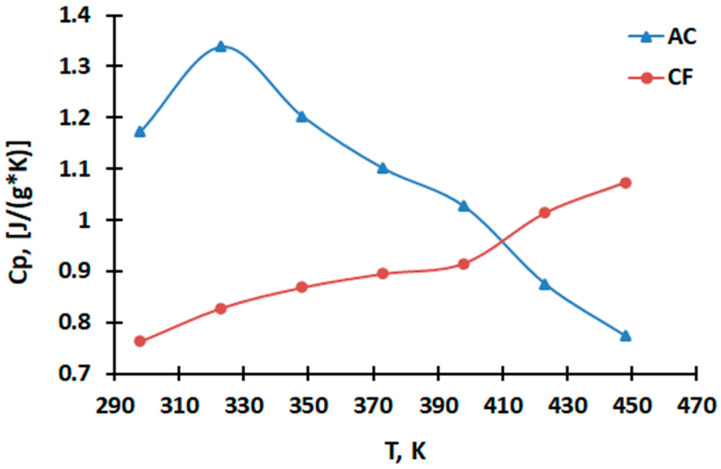
Graphical dependence of Cp~f(T) for coke fines (CF) and activated carbon (AC).

CF and AC are shown in Figure 1.

**Figure 2:**
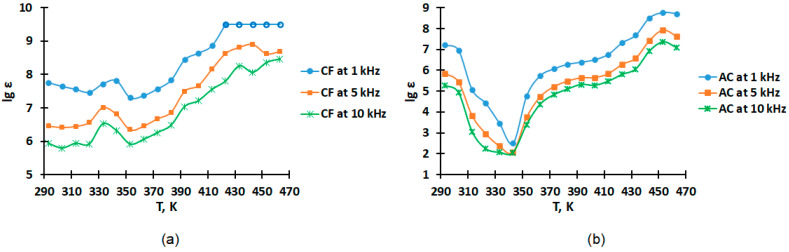
The temperature dependence of lgε in the range of 293–448 K at frequencies of 1, 5 and 10 kHz: (**a**) for coke fines (CF); (**b**) for activated carbon (AC).

**Figure 3:**
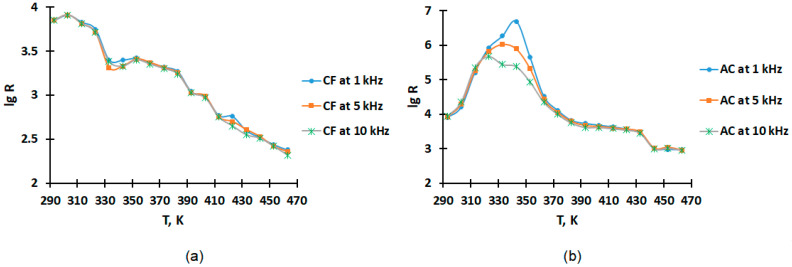
The temperature dependence of lgR in the range of 293–448 K at frequencies of 1, 5 and 10 kHz: (**a**) for coke fines (CF); (**b**) for activated carbon (AC).

From the experimental data presented in Figure 1, it can be seen that the AC sample at 323 K has a jump in heat capacity on the dependence curve C_p_~f(T), associated with a phase transition of the second kind. It should be emphasized that the phase transition at 323 K on the curve of the dependence of heat capacity on temperature can be attributed with some reservation to the transition temperature from semiconductor to metallic conductivity on the curve of the dependence of the relative permittivity on temperature (Figure 2) and electrical resistance on temperature (Figure 3), which also indicates the nature of this phase transition.

Based on the experimental data, it was found that the dependence of the specific heat capacity of the CF sample, taking into account the phase transition temperature, is described by the following equations [J/(g · K)]:

C_p_ = −(0.797 ± 0.046) + (6.61 ± 0.04) × 10^−3^T (298–323 K) (2)

C_p_ = (2.357 ± 0.137) − (3.76 ± 0.218) × 10^−3^T + (0.206 ± 0.012) × 10^5^T^−2^ (323–448 K) (3)

The change in the graphic dependence of C_p_~f(T) is probably due to the presence of carboxyl groups –COOH in activated carbon compared to the feedstock (coke fines), which was established by IR spectra in previous studies [28]. Carboxyl groups –COOH are thermally less stable than C–O [31]; therefore, the peak on the graph C_p_~f(T) is shifted towards lower temperatures.

### 2.2. Electrophysical Properties Measurement

Table 2 shows the results of electrophysical measurements of CF in the range of 293–463 K and at frequencies equal to 1, 5 and 10 kHz (where C, nF—capacitance; R, Om—electrical resistance; ε—relative permittivity).

The temperature dependences of lgε presented in Table 2 in the range of 293–448 K at frequencies of 1, 5 and 10 kHz for coke fines (CF) and activated carbon (AC) are shown as graphical dependences in Figure 2.

The results of studies of the temperature dependence of electrical resistance on temperature presented in Table 2 demonstrate that CF in the range of 293–463 K shows the semiconductor nature of the conductivity. The graphical dependence of lg R on temperature T is shown in Figure 3.

As can be seen from Figure 3, the electrical resistance of CF varies slightly at different frequencies of 1, 5 and 10 kHz and for AC a change in electrical resistance is observed in the range of 323–363 K.

As can be seen from the graphs of the dependences of the heat capacity on the temperature and (Figure 1) and the graphs of the dependences of the relative permittivity (Figure 2) and electrical resistance (Figure 3) on the temperature for activated carbon, there are coincidences of the temperatures of phase transitions. This may indicate that the change in the heat capacity of activated carbon at 323 K is associated with the transition of metallic conductivity to a semiconductor.

Calculation of the band gap width (Δ*E*) for CF in the range of 293–463 K: the calculation using values lgR = 3.85 at 293 K and lgR = 2.38 at 463 K is:

(4) $$\Delta E=\frac{2\times 0.000086173\times 293\times 463}{0.43\times (463-293)}\mathrm{lg}\frac{3.85}{2.38}=0.52\;eV$$

The width of the forbidden zone is 0.52 eV. This material can be attributed to narrow-band semiconductors.

The material has gigantic permittivity (ε) values. So, at 293 K and a frequency of 1 kHz has a value of 5.77 × 10^7^, and at 423 K it reaches a value of 1.01 × 10^9^ or more, i.e., more than a billion. At frequencies of 5 kHz and 10 kHz, its value decreases, but remains high enough (Table 1). The material is of interest for a microcapacitor. 

Electrophysical data in Table 2 show that AC exhibits metallic conductivity in the range of 293–343 K, and at 343–463 K demonstrates semiconductor conductivity.

Calculation of the band gap width (Δ*E*) for AC in the range of 293–403 K: the calculation was made according to the values lgR = 6.69 at 343 K and lgR = 2.97 at 463 K:

(5) $$\Delta E=\frac{2\times 0.000086173\times 343\times 463}{0.43\times (463-343)}\mathrm{lg}\frac{6.69}{2.97}=1.19\;eV$$

In this interval, the band gap width is 1.19 eV and it can be attributed to narrow-band semiconductors.

The material also has very large permittivity values (ε). At 293 K and 1 kHz, (ε) is equal to 1.63 × 10^7^, and at 463 K, 5.2 × 10^8^. The lowest value ε appears at 343 K, i.e., at the transition point of metallic conductivity to a semiconductor. With an increase in frequency from 1 to 10 kHz, the value of ε decreases. The material is of interest for capacitor technology.

The change in permittivity may be affected by moisture loss, the presence of functional groups C–O and O–H [32] as well as porosity. In the present study, in the case of the starting material, coke fines, the dielectric constant at different frequencies differs significantly from the dielectric constant of the resulting activated carbon (Figure 2), which may be due to low porosity and a higher water content compared to the resulting activated carbon. According to the results of IR spectroscopy in previous studies, the presence of absorption bands of weak intensity in coke fines (compared with the resulting activated carbon) corresponds to fluctuations of C=C bonds in polyaromatic hydrocarbons (at 1604.97 cm^−1^) and R–OH bonds of hydroxyl, carboxyl and phenolic compounds (at 3445.28 cm^−1^), which may indicate their low content.

Coal contains many aromatic and hydrocarbon components, and their reactions with steam at high temperatures can lead to the formation of various functional groups and compounds. In the present study, the activation of coke fines obtained from coal occurred by steam activation without the addition of chemical oxidants; therefore, carboxyl (-COOH) and hydroxyl (-OH) groups could be formed as a result of the thermal decomposition of organic functional groups that are already present in the structure of the coal material. As a result of steam activation of coke, hydroxyl (-OH) and carboxyl (-COOH) groups can be formed from coal. These functional groups can arise from aromatic structures and other carbon components of coal as a result of oxidation by water vapor. Aromatic structures in coal can be subjected to various reactions when exposed to steam. This can lead to the formation of phenolic compounds, as aromatic rings can undergo rupture, condensation and polymerization.

The interaction of coal material with water oxygen in a vapor medium can also contribute to the formation of hydroxyl and carboxyl groups.

The formation of carboxyl and hydroxyl groups under such conditions can occur through decarboxylation, when at high temperatures and exposure to steam, coal can lose carbon atoms in the form of carbon dioxide (CO_2_), which leads to the formation of carboxyl groups (-COOH) in the coal structure. The carbon material contains various functional groups, such as aromatic rings or alkyl groups [33]. During heat treatment, some of these functional groups can decompose, and some of the atoms from them can form carboxyl and hydroxyl groups.

It is important to note that the final number and type of functional groups in activated carbon will depend on many factors, including temperature, pressure, processing time and chemical composition of the initial carbon material. These functional groups can have a significant impact on the ability of activated carbon to adsorb various substances and determine its physico-chemical properties.

**Figure 4:**
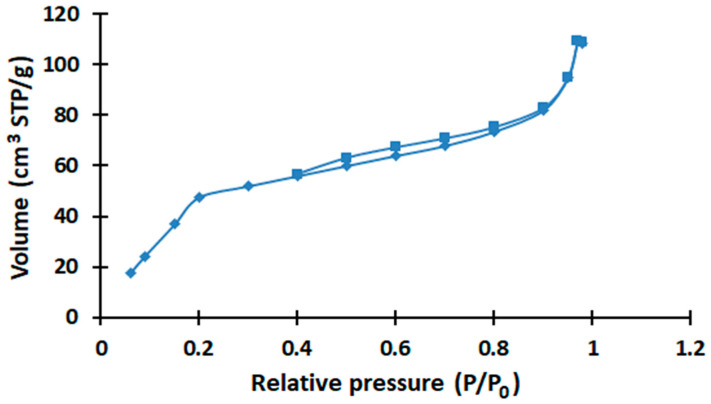
Adsorption isotherm of coal fines and activated carbon.

For activated carbon, there is a drop in the dielectric constant to a temperature of 343 K (Figure 2), which may be due to the removal of moisture as a result of heating. The presence of C=C bonds in polyaromatic hydrocarbons and functional groups C–O and O–H, as well as porosity greater than that of coke fines, can also contribute to the change in dielectric permittivity. In a previous study, based on the results of IR spectroscopy, it was found that there are high-intensity absorption bands in activated carbon corresponding to fluctuations in C=C bonds in polyaromatic hydrocarbons (at 1604.97 cm^−1^) and R–OH bonds of hydroxyl, carboxyl and phenolic compounds (at 3437.57 cm^−1^). These groups could be formed as a result of steam activation, since water vapor has high activity, which also contributes to the formation of a large number of mesopores [34]. In addition, depending on the content of oxidized carbon in activated carbon, its electrical resistance will change [33]. In this study, we found that the steam activation of coke fines led to an increase in the number of meso- and macropores; the results of the BET method are presented in Figure 4.

**Figure 5:**
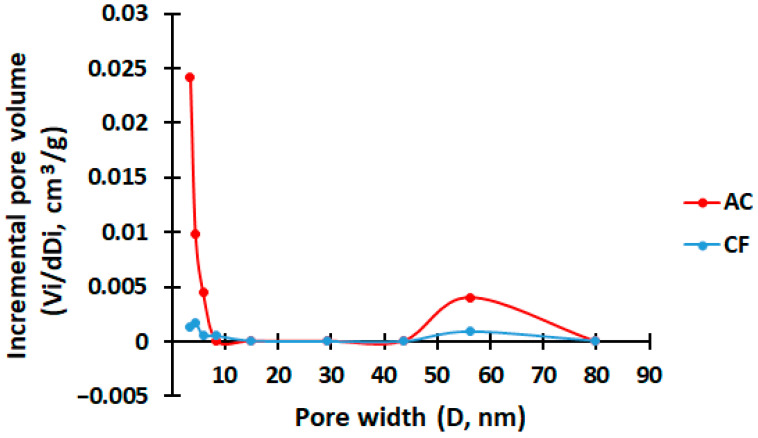
Pore size distribution of coke fines and activated carbon.

Based on the analysis of the graph of the adsorption/desorption isotherm of coke fines and the resulting activated carbon in Figure 4, it can be concluded that the type of hysteresis loop belongs to the H3 type. This type of loop is characterized by the fact that the values of volumes V on the desorption branch are less than on the adsorption branch over the entire P/P_0_ range. This behavior may be characteristic of porous materials with macro- or mesopores, where gases have difficulty escaping from deeper pores. According to the pore distribution graph in Figure 5, you can notice an increase in the number of meso- and macropores in the resulting activated carbon, which may affect the polarization processes [35].

## 3. Materials and Methods

In this article, the specific heat capacity and dielectric permittivity of activated carbon obtained by the steam activation of coke fines are studied. Activation was carried out in a stainless steel reactor at a temperature of 850 °C for 120 min [28]. 

### 3.1. Results of Heat Capacity Measurement

The temperature dependence of the heat capacity of the samples was measured on a serial calorimeter IT-S-400 (instrument-making plant, Aktobe, Kazakhstan) in the temperature range of 298.15–448 K.

The experiments were carried out in a monotonous, close to linear, heating of the sample at an average speed of about 0.1 K per second with temperature differences between the sample and the medium by 3–30 K. With such temperature differences, the temperature lag times on the heat meter are measured. In one experiment, the temperature dependence of the studied parameter is determined. The measuring circuit of the device provides a measurement of the temperature level from 100 to 400 °C at fixed points after 25 °C using a DC potentiometer and a switch built into the device. The heat meter was a heat flux converter, which provided flow measurements, equalized the surface temperature of the sample and made it possible to conduct calibration directly in the heat block to account for errors. The duration of measurements in the entire temperature range with the processing of experimental data was no more than 2.5 h. The limit of permissible error of the device according to the passport data is ±10.0%. The principle of operation of the device is based on the comparative method of a dynamic C-calorimeter with a heat meter. The test sample was placed in a metal ampoule of a measuring cell and heated continuously with a heat flow through a heat meter. At every 25 °C of heating, a time delay in the temperature of the ampoule in relation to the base temperature is measured using a microvoltammeter F-136 (Factory “Vibrator”, St. Petersburg, Russia). The calibration of the device was carried out on the basis of determining the thermal conductivity of the heat meter [36,37]. For this purpose, five experiments were conducted with an empty ampoule and the same number with a copper sample.

The thermal conductivity of the heat meter was determined by the formula:

(6) $$K_{T}=\frac{C_{Cu}}{\bar{\tau_{TM}\;}-\bar{\tau_{T}^{0}}}$$

where *C_Cu_* is the total heat capacity of the copper sample, J/(mol · K); $\bar{\tau_{TM}}$—the average value of the delay time on the heat meter in experiments with a copper sample, s; $\bar{\tau_{T}^{0}}$—the average value of the delay time in experiments with an empty ampoule, sec.

The total heat capacity of the copper sample was calculated by the formula:

C_Cu_ = C_ref_·m_s_ (7)

where *C_ref_* is the tabular value of the specific heat capacity of copper, J/(mol · K); *m_s_*—the mass of the copper sample, kg.

The value of the specific heat capacity of the test substance was calculated by the formula:

(8) $$C_{p}=\frac{K_{T}}{m_{0}(\tau_{T}-\tau_{T}^{0})}$$

where *K_T_* is the thermal conductivity of the heat meter; *m*_0_ is the mass of the substance under study; $\tau_{T}$ is the temperature delay time on the heat meter, s; $\tau_{T}^{0}$ is the temperature delay time on the heat meter in experiments with an empty ampoule, s. The mass of the sample of coke fines (CF) was 0.5859 g. The mass of the activated carbon (AC) sample was 0.4763 g.

At each temperature, five parallel experiments were conducted, the results of which were averaged and processed by methods of mathematical statistics.

At each temperature, for the averaged values of the specific heat capacity, the standard deviation ($\bar{\delta }$) was estimated according to [38]:

(9) $$\bar{\delta }=\sqrt{\frac{\sum_{i=1}^{n}\left(C_{i}-\bar{C}\right)}{n-1}}$$

*C_i_* is the measured value of the specific heat capacity,

$\bar{C}$—the arithmetic average of the measured values of the specific heat capacity.

The operation of the calorimeter was checked by determining the standard heat capacity of α-Al_2_O_3_. Its experimental value of [76.0 J/(mol · K)] is in satisfactory agreement with the reference data [79.0 J/(mol · K)] within ~4.0% [39].

To determine the error of the coefficients in the dependence equations C_p_~*f*(T), the values of the standard deviation for the temperature intervals under consideration were used.

The temperature dependence of the heat capacity of the coke fine (CF) sample was investigated by the method described above. At each temperature (after 25 K), five parallel experiments were carried out and their results were averaged by determining the standard deviation ($\bar{\delta }$) for the specific heat capacity.

### 3.2. Results of Electrophysical Properties Measurement

Measurements of electrophysical properties were carried out according to the methods [40,41].

The study of electrophysical properties (dielectric permittivity and electrical resistance) was carried out by measuring the electrical capacity of samples on a serial device LCR-800 (Taiwan) at an operating frequency of 1 kHz continuously in dry air in a thermostatic mode with a holding time at each fixed temperature.

Previously, plane-parallel samples were made in the form of disks with a diameter of 10 mm and a thickness of 5–6 mm with a binder additive (~1.5%). Pressing was carried out under a pressure of 20 kg/cm^2^. The resulting discs were fired in a laboratory furnace SNOL (AB “Utenos Elektrotechnika”, Utena, Lithuania) at 400 °C for 6 h. Then, they were thoroughly double-sided grinding.

The permittivity was determined from the electrical capacity of the sample at known values of the sample thickness and the surface area of the electrodes. To obtain the relationship between the electric induction D and the electric field strength E, the Sawyer–Tower scheme was used. Visual observation of the D (E hysteresis loop) was carried out on an oscilloscope C1-83 (Zolochiv Radio Factory, Zolochiv, Ukraine) with a voltage divider consisting of a resistance of 6 mOhm and 700 kOhm, and a reference capacitor of 0.15 UF. The frequency of the generator is 300 Hz. In all temperature studies, the samples were placed in an oven, the temperature was measured by a chromel –alumel thermocouple connected to a voltmeter B2-34 (JSC “Priboy”, Novorossiysk, Russia) with an error of ±0.1 mV. The rate of temperature change is ~5 K/min. The value of the dielectric constant at each temperature was determined by the formula:

(10) $$\varepsilon =\frac{C}{C_{0}}$$

where $C_{0}=\frac{\varepsilon_{0}∙S}{d}$ is the capacitance of the capacitor without the test substance (air).

The calculation of the band gap (Δ*E*) of the test substance was determined by the formula:

(11) $$\;\Delta E=\frac{2kT_{1}T_{2}}{0.43(T_{2}-T_{1})}\mathrm{lg}\frac{R_{1}}{R_{2}},$$

where *k* is the Boltzmann constant equal to 8.6173303 × 10^−5^ eV · K^−1^, *R*_1_ is the resistance at temperature *T*_1_ and *R*_2_ is the resistance at temperature *T*_2_.

## 4. Conclusions

In the range of 293–463 K, the temperature dependences of the electrophysical characteristics of coke fines and activated carbon were studied. By comparing the data of the dependences of the dielectric constant and electrical resistance on temperature, it was found that the jump in the heat capacity of activated carbon at 323 K is associated with a change in the type of conductivity.

In addition, if for coals obtained from vegetable raw materials, with an increase in temperature, there is a tendency to decrease the dielectric constant, then in this study it was found that activated carbon obtained in previous studies has an ambiguous character of changes in the dielectric constant associated with the presence of functional groups and mesopores, the increase of which occurred as a result of thermochemical exposure to water vapor.

Thus, coke fines can be considered as a promising raw material for obtaining materials for condenser technology. Materials with varying dielectric properties with temperature and frequency changes can be used to create capacitors with certain characteristics. For example, such materials can be used to create thermosensitive capacitors that change their capacitance depending on temperature. This can be useful in some applications where capacity monitoring is required depending on the ambient temperature. In addition, materials with variable dielectric properties can be used to create specialized capacitors with variable capacitance depending on frequency. Such capacitors can be used in various filtration schemes or to create devices with different frequency characteristics.

## Figures and Tables

**Table 1:** Experimental values of the specific heat capacity C_p_ [J/(g · K)] of the starting material (coke fines (CF) and the obtained activated carbon (AC) in the range of 298.15–448 K.

T, K	Coke Fines	Activated Carbon
	C_p_ ± δ	C_p_ ± δ
298	0.7629 ± 0.0180	1.1730 ± 0.0221
323	0.8280 ± 0.0215	1.3383 ± 0.0290
348	0.8681 ± 0.0180	1.2027 ± 0.0178
373	0.8949 ± 0.0251	1.1008 ± 0.0410
398	0.9152 ± 0.0159	1.0268 ± 0.0303
423	1.0133 ± 0.0170	0.8752 ± 0.0210
448	1.0729 ± 0.0297	0.7732 ± 0.0163

**Table 2:** The results of electrophysical measurements of CF and AC in the range of 293–463 K and at frequencies equal to 1, 5 and 10 kHz.

T, K	CF					AC				
	C, nF	R, O_M_	ε	lgε	lgR	C, nF	R, O_M_	ε	lgε	lgR
	1 kHz									
293	5729.7	7105	57,732,052	7.76	3.85	2267	7865	16,315,808	7.21	3.90
303	4425.3	8210	44,589,010	7.65	3.91	1299.3	15,870	9,351,182	6.97	4.20
313	3605.4	6702	36,327,756	7.56	3.83	16.189	157,300	116,514	5.07	5.20
323	2917.7	5618	29,398,539	7.47	3.75	3.6397	818,000	26,195	4.42	5.91
333	5162.6	2534	52,017,993	7.72	3.40	0.40742	1,836,000	2932	3.47	6.26
343	6548.3	2520	65,980,208	7.82	3.40	0.04409	4,917,000	317	2.50	6.69
353	2036.7	2658	20,521,645	7.31	3.42	8.1254	451,700	58,479	4.77	5.65
363	2321.3	2337	23,389,255	7.37	3.37	76.22	32,820	548,562	5.74	4.52
373	3697.1	2088	37,251,718	7.57	3.32	163.73	12,920	1,178,380	6.07	4.11
383	6777.9	1879	68,293,641	7.83	3.27	270.29	6681	1,945,302	6.29	3.82
393	27,544	1087	277,531,397	8.44	3.04	337.89	5388	2,431,826	6.39	3.73
403	43,275	966.6	436,035,842	8.64	2.99	449.5	4815	3,235,093	6.51	3.68
413	72,480	589.1	730,303,357	8.86	2.77	810.33	4260	5,832,020	6.77	3.63
423	>99,999	568.9	1,007,582,857˂	9.00˂	2.76	2917.5	3692	20,997,517	7.32	3.57
433	>99,999	397.4	1,007,582,857˂	9.00˂	2.60	6636.5	2864	47,763,504	7.68	3.46
443	>99,999	332.9	1,007,582,857˂	9.00˂	2.52	45,213	1047	325,402,138	8.51	3.02
453	>99,999	273.4	1,007,582,857˂	9.00˂	2.44	81,282	958.8	584,994,062	8.77	2.98
463	>99,999	241.1	1,007,582,857˂	9.00˂	2.38	72,280	938.5	520,205,837	8.72	2.97
**T, K**	**5 kHz**									
	**CF**					**AC**				
	**C, nF**	**R, O_M_**	**ε**	**lgε**	**lgR**	**C, nF**	**R, O_M_**	**ε**	**lgε**	**lgR**
293	287.04	7001	2,892,195	6.46	3.85	100.63	8377	724,243	5.86	3.92
303	263.37	8187	2,653,698	6.42	3.91	38.961	19,330	280,406	5.45	4.29
313	280.69	6494	2,828,213	6.45	3.81	0.93929	179,000	6760	3.83	5.25
323	366.19	5136	3,689,705	6.57	3.71	0.12581	642,000	905	2.96	5.81
333	1016.6	2033	10,243,190	7.01	3.31	0.03235	1,036,000	233	2.37	6.02
343	636.39	2158	6,412,221	6.81	3.33	0.01649	782,300	119	2.07	5.89
353	222.7	2555	2,243,909	6.35	3.41	0.7858	207,400	5655	3.75	5.32
363	285.97	2341	2,881,414	6.46	3.37	7.6724	27,130	55,219	4.74	4.43
373	471.15	2032	4,747,274	6.68	3.31	22.825	11,130	164,274	5.22	4.05
383	737.33	1769	7,429,285	6.87	3.25	42.547	6159	306,215	5.49	3.79
393	3122.9	1079	31,466,120	7.50	3.03	61.784	4649	444,665	5.65	3.67
403	4685.8	972.6	47,213,790	7.67	2.99	63.882	4355	459,765	5.66	3.64
413	14,462	557.7	145,718,090	8.16	2.75	97.663	3965	702,890	5.85	3.60
423	42,585	506	429,083,450	8.63	2.70	270.05	3593	1,943,575	6.29	3.56
433	64,673	405.5	651,640,577	8.81	2.61	537.41	2999	3,867,789	6.59	3.48
443	78,170	335.7	787,635,395	8.90	2.53	3755.8	1033	27,030,840	7.43	3.01
453	41,549	265.9	418,644,788	8.62	2.42	12,019	1103	86,501,853	7.94	3.04
463	48,901	231.7	492,723,020	8.69	2.36	6025.4	916.2	43,365,360	7.64	2.96
**T, K**	**10 kHz**									
	**CF**					**AC**				
	**C, nF**	**R, O_M_**	**ε**	**lgε**	**lgR**	**C, nF**	**R, O_M_**	**ε**	**lgε**	**lgR**
293	87.024	7032	876,848	5.94	3.85	26.717	8657	192,285	5.28	3.94
303	62.159	8057	626,310	5.80	3.91	12.159	22,400	87,509	4.94	4.35
313	87.164	6433	878,258	5.94	3.81	0.15926	224,000	1146	3.06	5.35
323	82.933	5166	835,627	5.92	3.71	0.0239	464,800	172	2.24	5.67
333	332.41	2401	3,349,340	6.52	3.38	0.0165	275,400	119	2.07	5.44
343	209.15	2160	2,107,381	6.32	3.33	0.01581	238,000	114	2.06	5.38
353	81.966	2511	825,884	5.92	3.40	0.35065	87,410	2524	3.40	4.94
363	113.54	2258	1,144,021	6.06	3.35	3.3075	22,460	23,804	4.38	4.35
373	182.13	2011	1,835,129	6.26	3.30	9.291	9992	66,868	4.83	4.00
383	302	1718	3,042,931	6.48	3.24	17.338	5607	124,783	5.10	3.75
393	1086	1080	10,942,459	7.04	3.03	28.703	4123	206,578	5.32	3.62
403	1606.6	935.3	16,187,988	7.21	2.97	26.412	4205	190,090	5.28	3.62
413	3506.4	563.1	35,330,239	7.55	2.75	42.101	3829	303,005	5.48	3.58
423	6223.9	450.9	62,711,577	7.80	2.65	87.57	3433	630,249	5.80	3.54
433	17,710	355.2	178,444,708	8.25	2.55	158.55	2803	1,141,099	6.06	3.45
443	11,488	324.3	115,752,276	8.06	2.51	1156.7	1008	8,324,877	6.92	3.00
453	22,214	260.2	223,826,694	8.35	2.42	3174.7	1038	22,848,609	7.36	3.02
463	28,432	211.1	286,478,823	8.46	2.32	1676.4	902.1	12,065,206	7.08	2.96

## Data Availability

Data are contained within the article.

## Sample Availability

Samples of the compounds are available from the authors.
